# Biosensing With Nanofluidics

**DOI:** 10.1002/EXP.20240433

**Published:** 2026-05-26

**Authors:** Qun Ma, Yan Xu

**Affiliations:** ^1^ Department of Chemical Engineering Graduate School of Engineering Osaka Metropolitan University Sakai Osaka Japan; ^2^ Japan Science and Technology Agency (JST) PRESTO Kawaguchi Saitama Japan; ^3^ Japan Science and Technology Agency (JST) CREST Kawaguchi Saitama Japan

**Keywords:** detection, nanochannels, nanopores, sensors, transducers

## Abstract

Nanofluidics, the study of fluid transport confined within nanochannels and nanopores, has emerged as a transformative technology across various domains, including sensing, separation, energy harvesting, materials synthesis, and bionic systems. Biosensing with nanofluidics, which involves the conversion of biomolecular information into fluidic signals through nanoscale fluid manipulation, is on the verge of revolutionizing the sensing paradigm, from DNA/protein sequencing, single‐molecule analysis, disease diagnosis, and precision medicine. In this review, we provide an overview of the current progress in nanofluidic biosensing, with a focus on in‐plane, out‐of‐plane, and free‐plane nanochannel and nanopore structures. We highlight the potential of nanofluidics in biosensing applications and discuss the current challenges faced in the development of nanofluidic‐based biosensing technologies. Furthermore, we explore future opportunities in this field, propose potential solutions to these challenges, and aim to contribute to the ongoing discourse in nanofluidic biosensing. Our insights are intended to pave the way for future advancements in this promising field.

## Graphical abstract

Nanofluidic biosensors employing in‐plane, out‐of‐plane, and free‐plane architectures transduce biomolecular information into fluidic signals through nanoscale manipulation of confined fluids, offering a quantitative and highly sensitive platform for biochemical detection.

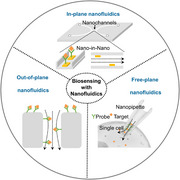

## 1 Introduction

Nanofluidics investigates fluid transport behaviors within nano‐ or subnano‐scale channels and pores, focusing on the precise manipulation and control of fluid dynamics in highly confined spaces [1, 2, 3]. Due to their unique characteristics, such as enhanced surface‐to‐volume ratios, fluid‐surface interactions, and pronounced molecular interactions, nanofluidics has revealed numerous unexpected behaviors and properties, fostering the exploration of new phenomena at the microscopic level [4, 5]. For instance, water molecules have been observed to flow through carbon nanotubes and graphene channels at rates significantly faster than those predicted by classical fluid dynamics [6, 7]. Additionally, water confined within nanometer‐scale channels exhibits dielectric properties that differ markedly from those of bulk water [8].  Ion transport in these confined spaces shows Coulomb blockade effects [9, 10], where the addition of a single ion to the channel is energetically unfavorable until a certain threshold is reached. Furthermore, the movement of one type of ion can drag along another type, resulting in coupled transport phenomena [11, 12]. These phenomena not only deepen our understanding of fundamental physics and chemistry at the nanoscale but also open up new possibilities for technological applications, including biochemical sensing, water treatment, energy conversion and storage, and next‐generation information technology [13, 14, 15, 16, 17, 18, 19, 20, 21, 22, 23, 24].

Nowadays, the development of biosensing technologies is of fundamental importance due to the increasing global demand for healthy living [25, 26, 27, 28, 29, 30, 31, 32, 33, 34, 35]. Nanofluidic‐based biosensing technology, as an emerging sensing technology, is on the verge of revolutionizing multiple domains, such as single‐molecule analysis, DNA/protein sequencing, disease diagnosis, and precision medicine [36, 37, 38, 39, 40, 41, 42, 43, 44, 45, 46, 47, 48, 49, 50, 51]. This technology offers a powerful tool for detecting and analyzing biomarkers with unparalleled sensitivity, specificity, accuracy, and spatiotemporal resolution, owing to its unique structural advantages [52, 53, 54, 55, 56, 57, 58, 59, 60, 61, 62, 63]. Nanofluidic biosensors with nanoconfined pore/channels are useful for the manipulation of biomolecules and for continuous operation, including sampling, separation, concentration, and transport. In turn, these features have provided opportunities to develop nanofluidic biosensors. For example, ultra‐high sensitivity is achieved due to the nanoconfinement effect, where even trace amounts of biomolecules can induce detectable changes in ionic current, enabling single‐molecule‐level sensing. Label‐free detection is enabled by the direct monitoring of ionic signal variations, eliminating the need for fluorescent or enzymatic labels. The system also supports multiplexed detection through the integration of multiple functionalized regions or recognition elements for simultaneous analysis of various biomarkers. Furthermore, the design allows for integration and miniaturization, facilitating the development of compact, portable biosensing devices. Over the past few decades, significant advancements have been made in nanofluidic biosensors across various domains, including medical diagnostics, genomics, proteomics, environmental monitoring, and pharmaceutical development. To expand their application scope, a diverse range of nanofluidic biosensors with varying compositions and structures have been developed. These include biological protein nanopores, membranes with single or multiple pores, carbon nanotubes, organic/inorganic nanochannel arrays, nanopipettes, and chip‐based nanofluidic devices. The evolution of these nanofluidic structures has significantly promoted the practical applications of nanofluidics in biosensing [15, 38, 39, 41, 51, 63, 64, 65, 66, 67, 68, 69, 70, 71, 72, 73, 74, 75, 76, 77].

Unlike traditional biosensors, nanofluidic biosensors operate by converting the chemical signals of biomolecules into fluidic signals, such as ion or molecular transport, which are then output as readable signals like optical or electrochemical signals [26, 78, 79, 80, 81]. Nanoconfinement is a key feature that enhances analyte‐fluid interactions, offering high sensitivity and spatiotemporal resolution. This allows for intrinsic signal amplification and detection sensitivity at the single‐molecule level. Consequently, nanofluidic biosensors provide significant advantages in single‐molecule and single‐entity analysis [36, 37, 38, 42, 43, 46, 50, 55, 57, 70, 80, 81, 82, 83, 84, 85]. Additionally, by functionalizing the channels with probes (DNA, enzyme, antibody, etc.) and regulating fluid‐interface interactions within nanofluidic channels, the selectivity of nanofluidic biosensors can be greatly improved [28, 41, 58, 86, 87, 88, 89, 90, 91, 92, 93, 94, 95, 96, 97]. These fluid‐interface interactions also facilitate in‐solution dynamic analysis of single molecules or particles, as well as single‐molecule chemical reactions [98, 99, 100, 101, 102, 103, 104, 105]. These characteristics enable nanofluidic devices to meet the demands of various biosensing applications, ranging from the analysis of trace target molecules in biological fluids to DNA/protein sequencing and in‐situ dynamic single‐molecule analysis.

**FIGURE 1:**
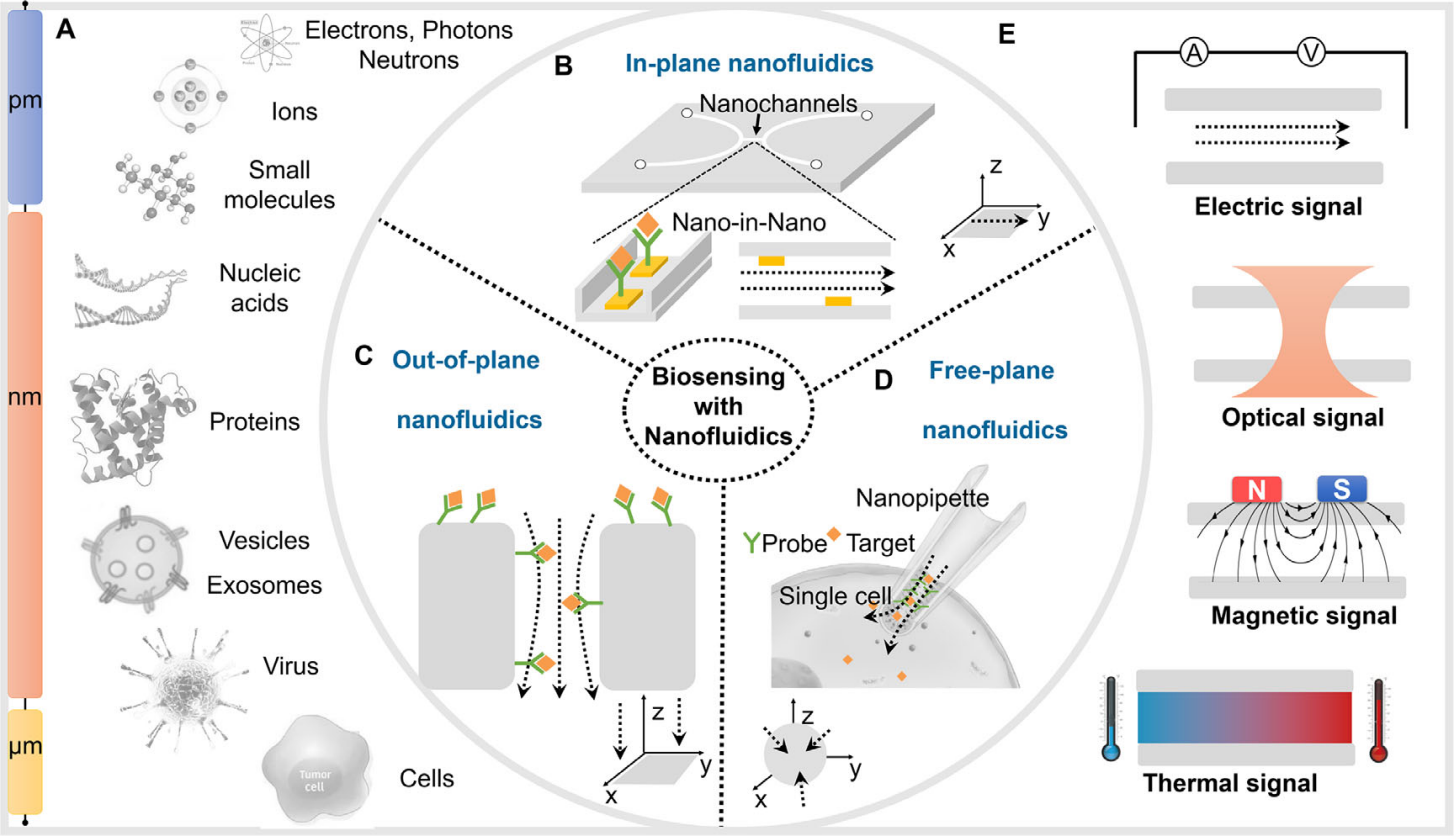
Biosensing with nanofluidics. (A) Biosensing process with nanofluidics including analyte recognition, signal conversion and transduction through nanofluidics, and signal processors. (B) Common bioanalytes including electrons, photons, neutrons, ions, small molecules, nucleic acids, proteins, vesicles, exosomes, virus, cells, and so on. Their sizes range from angstroms to micrometers, spanning six orders of magnitude. (C) Nanofluidics with in‐plane nanochannel/pores. (D) Nanofluidics with out‐of‐plane nanochannel/pores. (E) Nanofluidics with free‐plane nanochannel/pores. (F) Biosensing signals with nanofluidics, including electric, optical, magnetic, thermal signals, and so on.

Here, we discuss the recent advances in biosensing with nanofluidics, explore current challenges and potential solutions, as well as future opportunities in this field. (Figure 1). We provide a systematically overview of the current progress in biosensing with nanofluidics, focusing on in‐plane, out‐of‐plane and free‐plane nano channel/pore structures. We also discuss the challenges encountered in developing nanofluidics‐based biosensing. Furthermore, this review explores future opportunities in the field, highlights potential solutions to these challenges, and aims to contribute to the ongoing discourse in nanofluidic biosensing.

## 2 Nanofluidics With Different Structures

**FIGURE 2:**
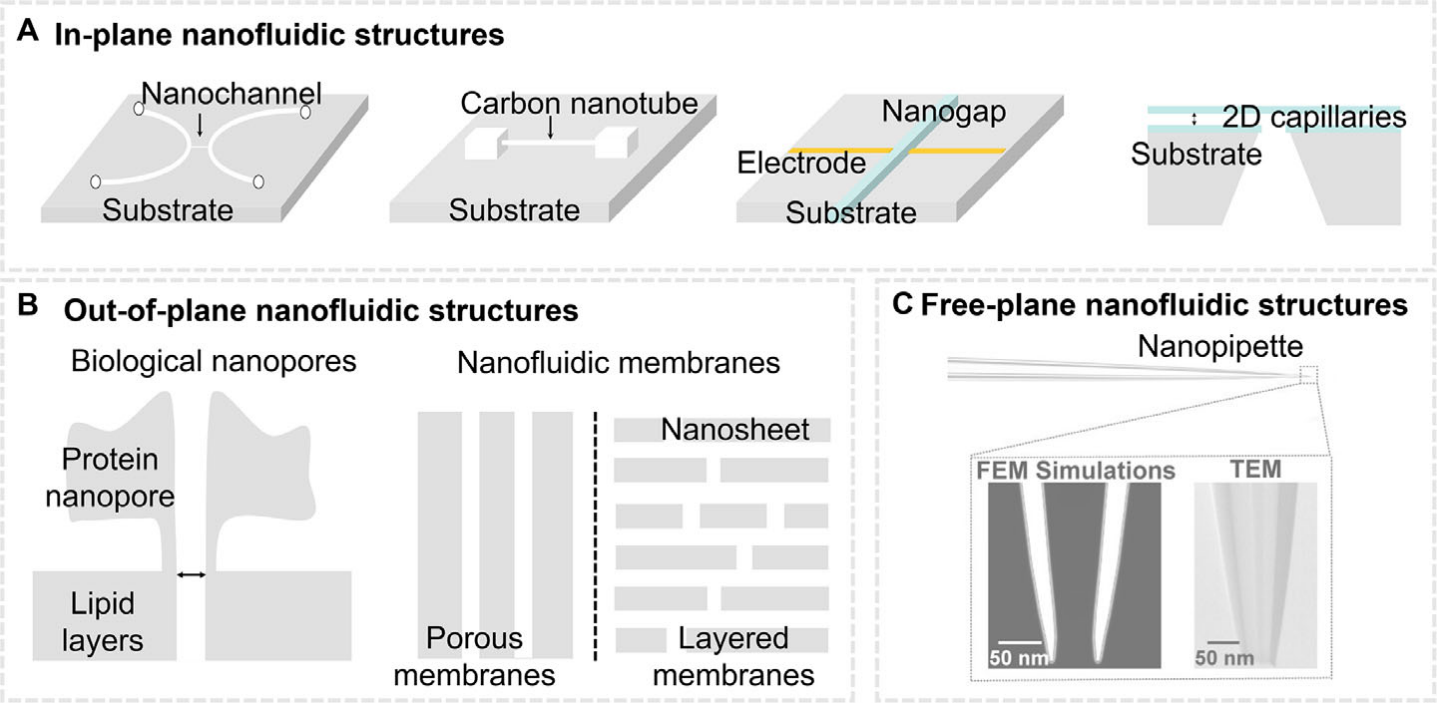
Nanofluidics with different structures. (A) Nanofluidics with in‐plane nanochannel/pores mainly includes chip‐based nanofluidic devices, carbon nanotube‐based nanofluidic devices, nanogap and two‐dimensional capillaries made by Van der Waals crystals, and so on. (B) Nanofluidics with out‐of‐plane nanochannel/pores. Typical samples include biological nanopores and solid‐state nanochannel/pore membranes. (C) Free‐plane nanofluidic structures. Typical sample is nanopipette‐based nanofluidic systems. Reproduced with permission [68]. Copyright 2016, American Chemical Society.

Significant efforts have been devoted to developing nanofluidic structures with finely tuned mass‐transport properties to broaden the biosensing applications of nanofluidic biosensors. To date, there are three main types of nanofluidic structures: in‐plane, out‐of‐plane, and free‐plane structures (Figure 2). Here, we classify nanofluidic structures by sensing dimensional mode. The term “sensing dimensional mode” refers to the spatial configuration and operational dimensionality of a nanofluidic biosensor during the detection process. It integrates both the structural geometry and the directional characteristics of sensing signal transmission, enabling classification into in‐plane, out‐of‐plane, and free‐plane modes. Each of these structures has distinct advantages suitable for different application scenarios. Specifically, in‐plane nanofluidic structures incorporate nanofluidic pores/channels that lie within the plane of a substrate [65, 74]. These primarily include chip‐based nanofluidic devices made from silicon, glass, or polymers, carbon nanotube‐based nanofluidics, nanogaps, and two‐dimensional (2D) capillaries assembled by van der Waals crystals [21, 39, 41, 73, 106, 107, 108, 109, 110, 111, 112, 113, 114, 115, 116, 117]. Out‐of‐plane nanofluidic structures, on the other hand, have nanofluidic pores/channels oriented perpendicular to the plane of the substrate. Typical examples include biological protein nanopores and artificial nanofluidic membranes with single or multiple pores/channels. Notably, nanofluidic membranes made from 2D nanolayered materials (such as graphene, MXene, MoS_2_, etc.) possess both in‐plane and out‐of‐plane nanofluidic structures [66, 118, 119]. This review focuses on their out‐of‐plane structures, as the in‐plane structures are not widely used in biosensing.

The third type is free‐plane nanofluidic structures. Unlike in‐plane and out‐of‐plane types, free‐plane nanofluidic structures allow for the flexible movement of nanofluidic biosensors according to specific needs. A typical example is nanopipette‐based nanofluidic systems [38, 43, 46, 47, 58, 67, 85, 89, 95, 120, 121, 122, 123, 124, 125, 126, 127, 128]. These nanofluidic structures are made in various materials (glass, etc.) and sizes and adapted to different biosensing applications. In‐plane nanofluidic structures are commonly used in in situ analysis by integrating other technologies, and they can be used for single‐particle analysis. Out‐of‐plane nanostructures easily integrate and are used for preparing high‐throughput nanofluidic biosensing devices. Free‐plane nanofluidic structures are suitable for in vivo single cell analysis at the single‐molecule level.

## 3 Biosensing With Nanofluidics

In contract to traditional biosensors (Table 1), biosensing with nanofluidics offers a unique platform for sensitive and rapid detection of biomolecules. This section primarily categorizes current nanofluidic biosensors into three types based on different nanofluidic structures. Focusing on the detection principles of each type of nanofluidic system, this paper mainly addresses the detection of electrochemical and optical signals owing to their convenient and sensitive ways. Other detection methods can be referred to in additional reviews and will not be elaborated on here. Similarly, the preparation and characterization of nanofluidic structures are detailed in other related reviews and will be omitted from this discussion. Instead, this paper will summarize representative advancements in the detection of optical and electrical signals based on different nanofluidic structures, to more clearly illustrate the advantages and broad application scenarios of nanofluidics in the field of biosensing (Table 2).

**TABLE 1:** The comparative analysis between nanofluidics and other emerging biosensing technologies.

Types	The size of sensing interface	The sensing environment	Sensitivity	Specificity	Sample type compatibility	Throughput and speed	Operational complexity and usability	Cost
Nanofluidics	nm	Nanoconfined	High	High	Liquid samples (blood, saliva, urine)	Medium to high	High	Moderate to high
Microfluidics	µm	Microconfined	Medium to high	Medium to high	Liquid samples	High	Moderate	Moderate
Nanoparticle‐based sensors	µm	Open	High	High	Liquid samples	Medium to high	Moderate	Moderate to high
Paper‐based biosensors	µm	Open	High	Medium to high	Liquid samples	High	Moderate	Low
FET‐based biosensors	nm	Open	High	High	Liquid samples	Medium to high	Moderate	Moderate
(SPR, FRET, LSPR) biosensors	nm	Open	High	High	Liquid samples	Medium to high	Moderate to high	Moderate to high

**TABLE 2:** The comparative analysis of in‐plane, out‐of‐plane, and free‐plane nanofluidic structures.

Types	Representative structures	Transport direction	Nanochannel size	Sensing signal	Sensitivity	Selectivity	Stability	Cost	Integrability	Applications
In‐plane nanofluidics	Chip‐based nanofluidic devices, carbon nanotubes, nanogaps, 2D capillaries, etc.	Horizontal	Length: 0.1µm–5 cm; Width: 10–500 nm; Height: 0.5–500 nm	Optical, electrochemical	High	Moderate	High	Medium	Easy	Continuous operation (sampling, separation, detection, and analysis); high‐throughput screening
Out‐of‐plane nanofluidics	Protein/DNA nanopores, membrane‐based nanofluidics	Vertical	Biological pores: 0.5–5 nm; solid‐state pores: 0.5–100 nm	Ionic current	High	High	Moderate to high	Solid‐state pores: low; Biological pores: high	Moderate	Single‐molecule analysis, sequencing, multi‐analyte analysis
Free‐plane nanofluidics	Nanopipettes, etc.	Free	Tip aperture: ≈5–200 nm;	Ionic current	High	High	Low to moderate	High	Moderate	Single‐cell analysis, in situ detection

### 3.1 Biosensing With In‐Plane Nanofluidics

There is a wide range of nanofluidic devices with in‐plane nanopore/channels, where the nanopore/channels lie within the plane of a substrate, typically chip‐based nanofluidic devices. For example, nanofluidic transistors based on carbon nanotubes on the silicon substrate have been used for the voltage‐gated ion/molecule transport through nanochannels [110, 111]. Nanofluidic biosensors based on nanogap electrodes were developed for the fast real‐time label‐free biomolecule analysis [129, 130, 131, 132]. Nanofluidic memristors using in‐plane nanofluidic structures that were assembled by Van der Waals crystals have been constructed for long‐term memory and synapse‐like dynamics [21].

**FIGURE 3:**
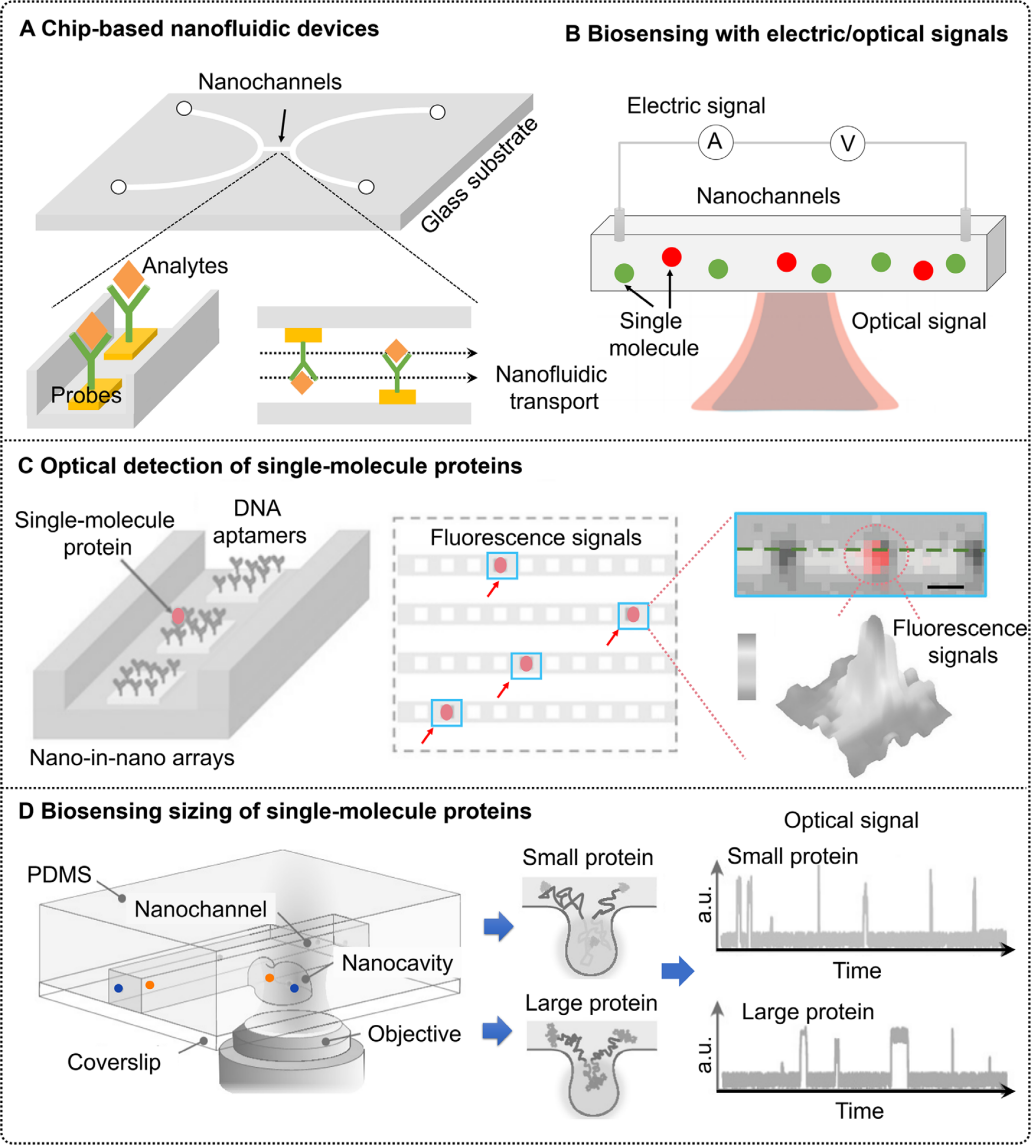
Chip‐based nanofluidic devices with in‐plane structures for biosensing. (A) Chip‐based nanofluidic devices as a typical sample of in‐plane nanofluidics. (B) Biosensing principle of chip‐based nanofluidic devices with electric and/or optical detection signals. (C) Chip‐based nanofluidic devices to enable stochastic capture of single proteins at normal concentrations using optical output signals. Reproduced with permission [41]. Copyright 2023, Wiley‑VCH GmbH. (D) Chip‐based nanofluidic devices to enable stochastic capture of single redox molecules using electrochemical signals. Reproduced with permission [146]. Copyright 2023, American Chemical Society.

In comparison with popular microfluidic devices, chip‐based nanofluidic devices are at a nascent stage but possess great potential owing to their special phenomena and nanoscale effects. Chip‐based nanofluidic devices are miniaturized platforms that integrate nanofluidic channels and structures onto a single chip, typically fabricated using materials such as silicon, glass, or polymers [62, 76, 133, 134]. Their unique advantages including planar, transparent, in‐plane, and solid‐state characteristics enable them to be easily coupled with a variety of microscopes and exhibit flexibility superior to that of other nanofluidic geometries. These devices leverage the principles of nanofluidics to manipulate and control fluids at the nanoscale, enabling precise and efficient analysis of minute sample volumes [39]. Hence, chip‐based nanofluidic devices have been widely used to visualize biosensing (Figure 3A,B). The key features and advantages of chip‐based nanofluidic devices include:

(1) Integration and miniaturization: The integration of multiple functions onto a chip facilitates the development of compact, portable, and automated systems, which are ideal for point‐of‐care diagnostics and on‐site analysis; The integration of such nanofluidic features into mass spectrometers would open up future avenues for the potential of mass spectrometry to explore unknown subcellular matter at a nanoscale [70].

(2) Versatility and multifunctionality: These devices can be designed to perform a wide range of analytical tasks, including sample preparation, separation, detection, and analysis, making them versatile tools for various applications in biosensing, genomics, proteomics, and environmental monitoring.

(3) Low sample and reagent consumption: The nanoscale operation reduces the volume of samples and reagents required, which is particularly advantageous when dealing with scarce or expensive materials.

In addition, nanofluidic devices fabricated using glasses show the features of planar, transparent, and solid‐state characteristics that can be easily coupled with a variety of microscopes and exhibit flexibility. These advanced features suggest that chip‐based nanofluidic devices hold promise as tools for detecting and regulating single small molecules in fluidic conditions [77, 112, 135, 136, 137, 138, 139, 140, 141, 142, 143, 144, 145]. For example, Xu et al. reported a chip‐based nanofluidic device featuring a nanochannel aptamer nanoarray (NANa) that enables the immunologically specific capture of single proteins at physiological concentrations [41, 88] (Figure 3C). This device utilizes a high‐density nanofluidic aptamer nanoarray produced through site‐specific molecular self‐assembly within tiny, closed square nanochannels featuring nano‐in‐nano functional patterns. The resulting nanofluidic aptamer nanoarray demonstrates a remarkable ability to selectively capture target proteins within the nanochannels. Consequently, the device facilitates the stochastic capture of individual proteins even at normal concentrations, in alignment with the Poisson distribution. In addition, nanofluidic devices have emerged as powerful tools for the analysis of nanoscale matter. Lemay et al. pioneered the detection of single molecules using electrochemical methods within a chip‐based nanofluidic platform. Their approach, based on the anticorrelated current signals generated by molecules traversing nanofluidic channels, offers a label‐free alternative to fluorescence‐based techniques [135, 136]. Moreover, nanofluidic channels integrated with micropatterned lipid membranes have shown promise for sensitive membrane protein analysis [137], while nanotrap geometries have demonstrated the ability to extend observation timescales for nanoscale particles, including biomacromolecules and colloids [138]. These nanotrap structures effectively confine particles, leading to significant increases in residence times within the detection volume. Knowles et al. introduced a microfluidics/nanofluidics‐based approach for producing monodisperse water‐in‐oil emulsions with precise size control [140]. Han et al. developed a nanofluidic device capable of continuous, high‐sensitivity, and high‐resolution monitoring of biologic purity and bioactivity. The device employed periodic and angled nanofilter arrays for size‐based analysis of biologics [141]. The versatility of nanofluidic devices extends to particle sizing, as evidenced by the work of nanofluidic‐based approaches have been developed for sizing nanoscale particles and single biomolecules in solution [146] (Figure 3D). Additionally, Jacobson et al. employed resistive‐pulse measurements to characterize extracellular vesicles derived from bovine milk and human breast cancer cells [147]. The devices were fabricated in a planar configuration featuring three nanopores arranged in series, enabling precise measurements of particle volume and diameter.

These collective advancements underscore the potential of nanofluidic technologies to revolutionize fields such as analytical chemistry, biochemistry, and materials science.

### 3.2 Biosensing With Out‐of‐Plane Nanofluidics

Here, the typical types of out‐of‐nanofluidics including biological nanopores and membrane‐based nanofluidics, were mainly discussed. The key features of out‐of‐plane nanofluidics are as follows:

(1) Vertical orientation: Channels and structures extend vertically from the substrate, allowing for three‐dimensional (3D) fluidic pathways. This orientation is crucial for the functionality of biological nanopores and membrane‐based systems;

(2) Enhanced surface area: The out‐of‐plane design increases the surface area available for interactions between the fluid and the channel walls. This is beneficial for applications requiring high surface‐to‐volume ratios, such as biosensing and chemical analysis;

(3) Ease of fabrication and scalability: Out‐of‐plane nanofluidic devices offer significant advantages in terms of ease of fabrication, scalability, and integration with existing technologies.

These benefits, combined with their enhanced performance, cost‐effectiveness, versatility, and robustness, make them highly attractive for a wide range of applications. This section mainly discusses biosensing with out‐of‐plane nanofluidics.

Biological nanopores, such as those derived from proteins like alpha‐hemolysin or engineered variants like MspA, are embedded in lipid bilayers or synthetic membranes [148, 149, 150, 151, 152, 153, 154]. These nanopores can detect various biomolecules, including DNA, RNA, proteins, and small molecules, by monitoring changes in ionic current as these molecules pass through or interact with the pore. Biological nanopores can achieve label‐free detection of individual molecules, providing high sensitivity and enabling the analysis of minute quantities of sample with high temporal resolution, allowing for single‐molecule analysis, DNA/protein sequencing, and the real‐time monitoring of molecular interactions and dynamics [155, 156, 157, 158, 159]. Kilobase‐length polymers, such as single‐stranded DNA or RNA, along with small molecules like nucleosides, can be identified and detected without necessitating amplification or labeling. This unique analytical capability facilitates rapid and cost‐effective DNA sequencing [160]. For example, Wu et al. discovered that when a peptide with phenylalanine at the N‐terminus was combined with a guanidine ring molecule, the resulting nanopore exhibited ultra‐high resolution for specific amino acids, enabling effective differentiation among the 20 standard amino acids [161]. Through a combination with carboxypeptidase and coupling reactions, nanopore technology successfully enabled the first‐ever determination of short peptide sequences. Bayley and his colleagues were pioneers in employing protein nanopores for sequencing and single‐molecule analysis, establishing a foundational framework that has facilitated numerous innovations in the field. A notable advancement from their research is the development of a “molecular hopper,” which is capable of translocating single‐stranded DNA through a protein nanopore [162]. This molecular hopper operates by sequentially forming and breaking chemical bonds that link simple strands to a nanoscale track. Remarkably, this process can be regulated—turned on, off, or reversed—by applying small electrical potentials, positioning it as a promising candidate for integration into nanopore DNA sequencing devices. The molecular hopper achieves sub‐nanometer (0.7 nm) hops, with each movement meticulously monitored in real time, allowing for precise observations at the single‐molecule level. This innovative approach not only advances the development of nanopore‐based DNA sequencing but also contributes to the growing field of biological machines designed to manipulate molecular processes at the single‐molecule scale. Moreover, biological nanopores hold significant promise for the investigation of single‐molecule proteins, providing vital support for the advancement of protein sequencing and proteomics. Long et al. report a novel strategy for investigating temporal changes in molecules within complex systems through the application of nanopore single‐molecule interfaces [152]. By employing a meticulously designed nanopore interface, an independently constructed weak current measurement device, and a targeted methodology for rapid single‐molecule quantification, they sequenced a series of angiotensin peptides, each differing by a single amino acid, within a multi‐component system. This innovative approach not only allowed for the real‐time monitoring of dynamic evolution processes of angiotensin peptides but also elucidated their quantitative evolution pathway. Moreover, it shed light on the crosstalk effects inherent in the renin‐angiotensin system and proposed a model detailing the impact of the SARS‐CoV‐2 spike protein and its variants on this biological system. Winterhalter et al. employed an electrostatically asymmetric nanopore as a chiral biosensor, enabling the identification of single amino acid chiral and positional isomers [163]. This orientation subsequently affects the characteristic single‐molecule current that flows through the pore. By analyzing the specific amplitudes of current blockades and their corresponding residence times, precise identification of chiral and positional isomers can be achieved. Furthermore, nanopores have been utilized for single‐molecule analysis of metal ion‐chelator chemical reactions, as well as for real‐time monitoring of disulfide bond formation and cleavage [164]. These investigations have illustrated the capability of nanopores to unveil intricate details of chemical processes at the single‐molecule level. Additionally, biological nanopores have been employed for the real‐time measurement of protein‐protein interactions at single‐molecule resolution, further underscoring their versatility and precision [165]. Collectively, these studies highlight the potential of biological nanopores as powerful analytical tools at single‐molecule level.

**FIGURE 4:**
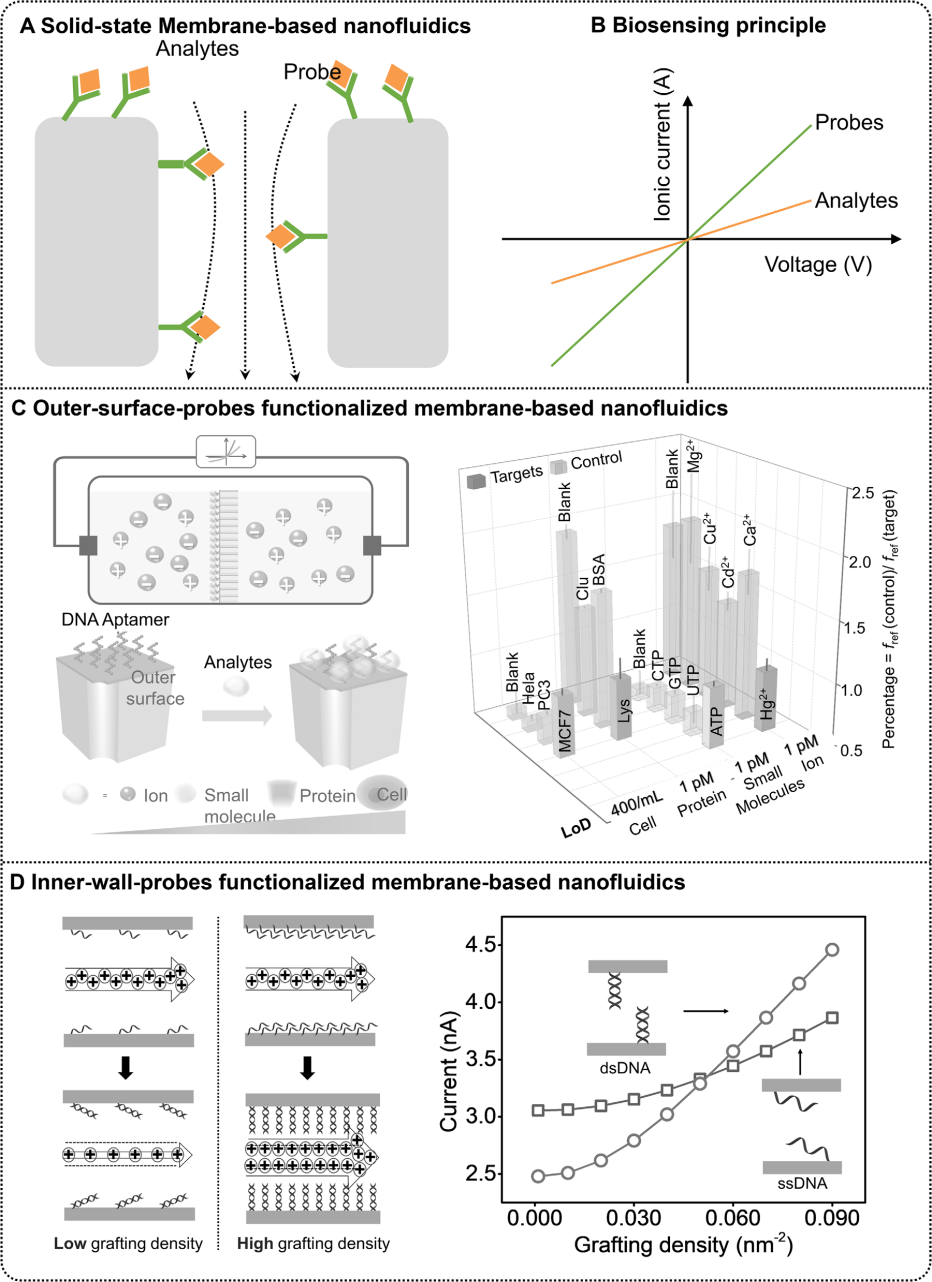
Solid‐state membrane‐based nanofluidics with out‐of‐plane nanochannel/pores for biosensing. (A) Membrane‐based nanofluidics as a typical sample of out‐of‐plane nanofluidic structures. (B) Biosensing principle of membrane‐based nanofluidics with steady ion current. (C) Membrane‐based nanofluidics for universal detection of biomarkers using the outer surface as sensing interfaces. Reproduced with permission [93]. Copyright 2021, The Author(s), published by Springer Nature. (D) Biosensing with membrane‐based nanofluidics using inner walls as sensing interfaces. Reproduced with permission [185]. Copyright 2021, American Chemical Society.

Inspired by biological ion channels, solid‐state membrane‐based nanofluidics have made great progress in recent years owing to the development of advanced fabrication and characterization technologies, and have been proven to be a versatile new tool for understanding the functionality of biological protein channels and development of novel nanofluidic‐based devices, such as smart gating devices, biochemical sensors, and so on [18, 53, 71, 166, 167, 168, 169, 170]. Solid‐state membrane‐based nanofluidics, as another out‐of‐plane nanofluidic structure leverages the unique properties of nanoscale fluidic channels and pores to manipulate and analyze biological and chemical entities with unprecedented precision, offering robust, stable, and highly controllable environments for the detection of biological molecules (Figure 4A). A wide range of membrane materials, including polymeric membranes, inorganic membranes, and two‐dimensional layered materials, have been synthesized to build membrane‐based nanofluidics [171, 172, 173, 174, 175, 176, 177]. As biomolecules translocate through membranes, they cause changes in the ionic current, which can be measured to detect and quantify the presence of the analytes (Figure 4B). In addition, integration with optical detection systems, such as fluorescence or surface plasmon resonance, can provide additional sensitivity and specificity [178, 179, 180]. Meanwhile, protein channels with explicit structure and function division have been demonstrated to precisely control the state of biological channels and regulate the life information transport. This inspires researchers to construct precise division of outer surface and inner walls of membrane‐based nanofluidics [92, 93, 158, 174, 181, 182, 183]. Moreover, by virtue of the special advantages (easy programmable structure, adjustable functional groups, abundant charge units, and easy surface assembly) of DNA nanotechnology, one can precisely control the physicochemical properties of OS and IW of membranes [1, 14, 58, 75, 86, 87, 90, 91, 95, 126, 128, 184, 185, 186, 187, 188, 189, 190, 191, 192, 193]. The integration of membrane‐based nanofluidics and DNA nanotechnology opens up new avenues for biosensing. For instance, Xia et al. were the first to experimentally demonstrate the synergistic enhancement effect resulting from the outer surface in regulating ion gating on the inner walls of membrane‐based nanofluidics through the employment of designed DNA structures as functional molecules [182]. This effectively reduced false signals associated with ionic gating, particularly in complex environments. Furthermore, they expanded on this concept by demonstrating that the DNA‐modified outer surface can independently regulate ion transport in membrane‐based nanofluidics, even in the absence of inner walls, thereby achieving multiscale target sensing [93] (Figure 4C). Nanofluidic biosensing leverages the inner walls of nanochannels as highly confined sensing interfaces, where probe molecules directly modulate ion transport. The current study demonstrates that tuning the grafting density of DNA probes on these inner walls reshapes the balance between steric hindrance and electrostatic interactions, leading to a switchable ionic signal from “off” to “on.” This reveals how nanometric confinement amplifies interface‑dependent sensing behaviors and provides a mechanistic basis for optimizing inner‑wall‑regulated nanofluidic biosensors [185] (Figure 4D). Omar et al. reported the development of a nanofluidic‐based biosensor featuring inner walls as sensing interfaces, enabling the direct detection and differentiation of infectious from non‐infectious human adenovirus and SARS‐CoV‐2, as well as a range of other virus types, without the need for sample pretreatment [87]. This design allows for the effective capture and analysis of the target viruses, achieving detection limits as low as 1 plaque‐forming unit (pfu)/mL for human adenovirus and 10^4^ copies/mL for SARS‐CoV‐2. The strong confinement provided by the nanopore facilitates greater interaction between the aptamers and the viral particles, improving the overall sensitivity and enabling accurate detection of low viral loads, which is critical for diagnostics and monitoring infectious diseases.

### 3.3 Biosensing With Free‐Plane Nanofluidics

**FIGURE 5:**
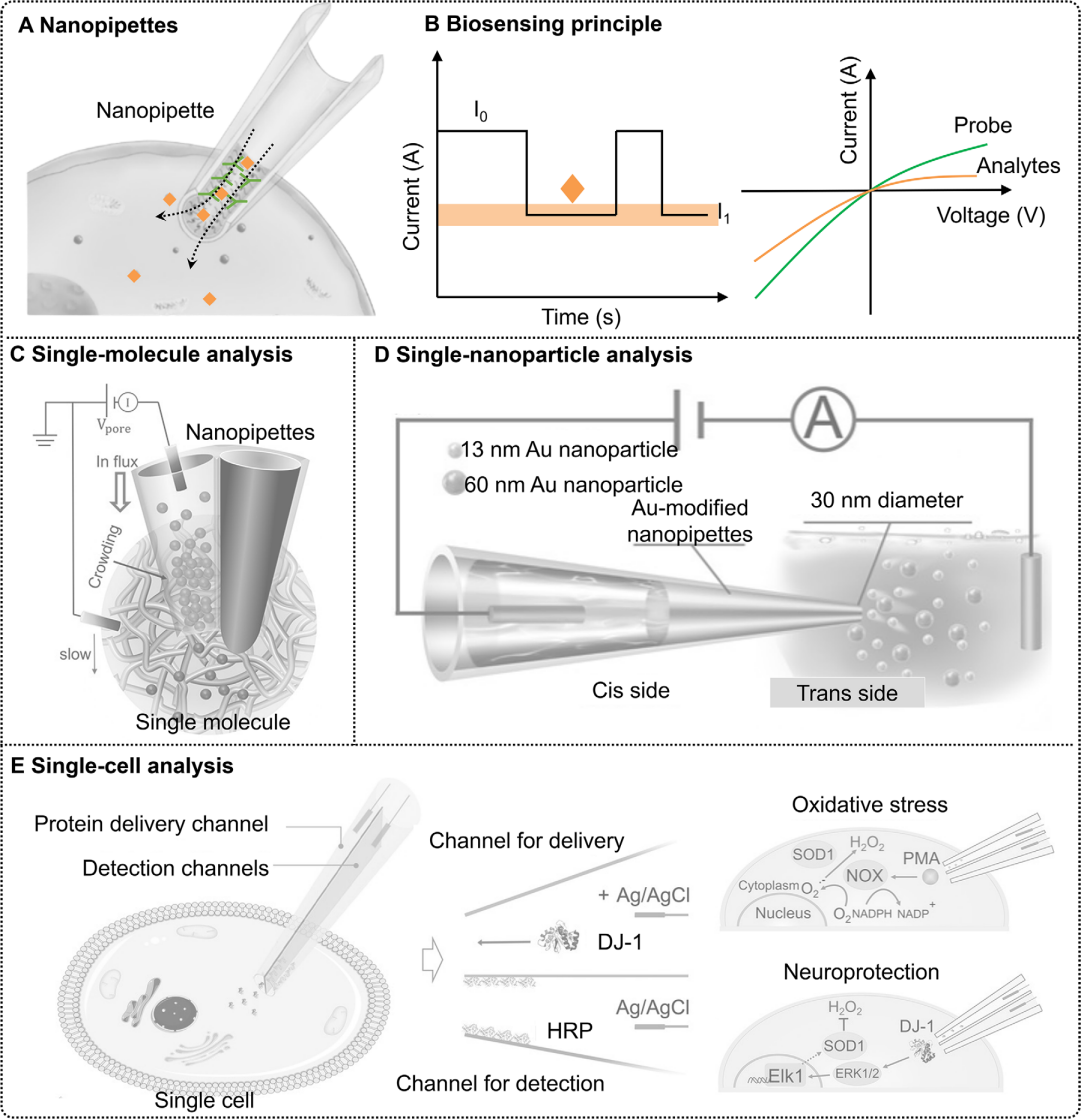
Nanopipette‐based nanofluidics with free‐plane structures for biosensing. (A) Nanopipette‐based nanofluidics as a typical sample of free‐plane nanofluidic structures. (B) Biosensing principle of nanopipette‐based nanofluidics with ion blockade current and/or steady ion current. (C) Nanopipette‐based nanofluidics for single‐molecule analysis. Reproduced with permission [203]. Copyright 2023, American Chemical Society. (D) Nanopipette‐based nanofluidics for detection of the dynamic interactions of every single particle in the mixture. Reproduced with permission [206]. Copyright 2018, Wiley‑VCH Verlag GmbH & Co. KGaA, Weinheim. (E) Nanopipette‐based nanofluidics for electrochemical single‐cell protein therapeutics. Reproduced with permission [207]. Copyright 2023, Wiley‑VCH Verlag GmbH & Co. KGaA, Weinheim.

Limited by in‐plane and out‐of‐plane nanofluidics, in many application scenarios, we need to be able to move nanofluidic devices freely to achieve biosensing sometimes. We define such freely movable nanofluidic devices as free‐plane nanofluidics (Figure 5A). Nanopipettes are a representative free‐plane nanofluidics structure, which not only maintains the advantages of traditional nanofluidic structures, but also has free mobility and flexibility [38, 43, 46, 47, 58, 67, 68, 69, 85, 89, 95, 120, 121, 122, 123, 124, 125, 126, 127, 128, 177, 194, 195, 196, 197, 198, 199, 200, 201]. Nanopipettes are ultra‐fine pipettes with nanoscale tips, typically made from materials like borosilicate glass or quartz. For the fabrication of nanopipettes, laser pulling is a widely used and cost‐effective method. The ability to modify the inner wall to exhibit good selectivity is particularly advantageous for detecting and quantifying important biomolecules within the complex intracellular environment. The working principle is based on the affinity‐based binding between the probes tethered to the surface and their targets, causing a change in the ionic current due to a partial blockade or an altered surface charge (Figure 5B). Nanopipettes are capable of manipulating and sensing small volumes of fluids with high precision, and operate at the nanometer scale, making them essential tools in nanofluidics biosensing, including single‐molecule, single‐cell, and single‐nanoparticle analysis.

Using the principle of current blockade, nanopipettes can be used for single‐molecule analysis [202, 203, 204, 205]. For example, He et al. employed nanopipettes that leverage a strong nanoconfinement effect, effectively slowing the exit motion of small molecules and enabling their enrichment and trapping at the nanopipette tip [203] (Figure 5C). This methodology exploits the synergistic effects of strong nanoconfinement and viscous hydrogel to induce controllable crowding of small biomolecules near a nanopipette tip. The methodology exhibits exceptional sensitivity and efficiency, enabling the detection of even minute biomolecules such as nucleoside triphosphates (less than 1 kDa), short peptides (a few kDa), and the hormone insulin (5.8 kDa). Remarkably, detection rates exceeding 100 events per second can be achieved, even with nanopores that are more than 15 times larger than the target molecules. Moreover, the crowded environment created by this approach facilitates specific intermolecular interactions, allowing for the observation of complex formations like nucleoside triphosphate pairs and quadruplexes. This methodological advancement not only enhances the sensitivity and efficiency of biomolecule detection but also provides a powerful tool for investigating molecular interactions and complex formations at a higher resolution.

Nanopipettes have also been successfully utilized for single‐nanoparticle analysis. For instance, Long et al. developed nanopipette biosensors featuring a 30 nm conductive nanopore electrode with precise and well‐defined morphology specifically for single‐nanoparticle assessment [206] (Figure 5D). A novel 30 nm confined nanopore electrode was developed to directly detect intrinsic collision events of single nanoparticles with varying sizes. This precise fabrication method enables highly reproducible control over nanopore dimensions. The detection mechanism, based on capacitance, offers exceptional current resolution, achieving a remarkable precision of 0.6 ± 0.1 pA. Moreover, the system boasts a temporal resolution of 0.01 ms, enabling the discrimination of microsecond collision events of single nanoparticles from the recorded current traces.

The pursuit of single‐cell technologies has gained significant momentum due to their potential to enhance our understanding of cellular physiology and to facilitate precise pathological examinations and therapeutic interventions. Consequently, developing technologies for precise single‐cell analysis is fundamental. Nanopipettes can be directly inserted into living cells, enabling real‐time detection of intracellular substances through the electric field at the nanopipette tip. This minimally invasive approach preserves cell viability, making it an invaluable tool for studying cellular processes without compromising cellular integrity. Researchers can leverage these tools to gain deeper insights into cellular functions, interactions, and responses, ultimately advancing our understanding of fundamental biological processes and disease mechanisms. For example, Xu et al. reported a nanopipette tool that exhibits excellent selectivity, stability, and high recyclability for the electrochemical analysis of biomolecules within single cells [207] (Figure 5E). This dual‐lumen nanopipette enables the precise delivery of cytosolic proteins, such as DJ‐1, into neural PC‐12 cells and the simultaneous monitoring of cellular responses. By delivering DJ‐1, an upregulation of the antioxidant protein was observed, leading to protective effects against oxidative stress induced by phorbol myristate acetate. The second lumen, dedicated to ionic evaluation, allowed for the real‐time monitoring of cytosolic hydrogen peroxide levels, a key indicator of cellular oxidative stress. This innovative approach offers significant potential for advancing electrochemical techniques in protein therapeutics.

## 4 Future Challenges and Perspectives

Biosensing with nanofluidics represents one of the most promising frontiers of biosensing technologies in the realms of precision medicine, disease diagnosis, and health management. The development of various nanofluidic structures has rendered nanofluidic technology adaptable to diverse biosensing scenarios. Despite its recent advances, some challenges still need to be addressed. For example, the ultrasensitive detection of cancer‐associated biomarkers (proteins, antibodies, hormones, cytokines, DNA or RNA, etc.) in complex biofluids (tears, breath, digestive fluid, saliva, sweat, urine, etc.) is challenging, as most biomarkers are present at very low concentration and with transient heterogeneity. Detecting low‐concentration biomarkers in biologically complex samples requires the development of nanofluidic biosensors with high sensitivity and specificity to meet the demands of practical sample analysis. In addition to the low concentration and transient heterogeneity of target biomarkers, another significant challenge in practical applications is the nonspecific adsorption of non‐target species present in complex biofluids. These interfering molecules can adhere to the inner surfaces of nanochannels, leading to channel blockage, reduced sensitivity, and false‐positive signals. This phenomenon not only compromises sensor accuracy but also affects long‐term device stability and reliability. Addressing this issue requires advanced surface modification strategies, such as the use of anti‐fouling coatings (e.g., polyethylene glycol, zwitterionic polymers) and selective molecular recognition layers, to minimize unwanted interactions while preserving the capture efficiency of target analytes. Traditional single‐biomarker analysis sometimes fails to accurately reflect disease status and can lead to misdiagnosis. Therefore, it is essential to develop nanofluidic biosensors capable of simultaneously and precisely analyzing multiple biomarkers, enabling early and accurate diagnosis of major diseases such as cancer. In clinical auxiliary diagnosis, rapid detection and data analysis are also crucial, in addition to meeting requirements for sensitivity, specificity, and accuracy. Point‐of‐care‐testing represents an important future application scenario for biosensors; however, developing nanofluidic biosensors with high stability and real‐time monitoring capabilities remains a significant challenge. Moreover, biocompatibility and safety are among the key issues that still need to be addressed. Finally, for the development of more smart nanofluidic biosensors, the integration of therapeutic functions alongside disease detection still presents a major challenge.

**FIGURE 6:**
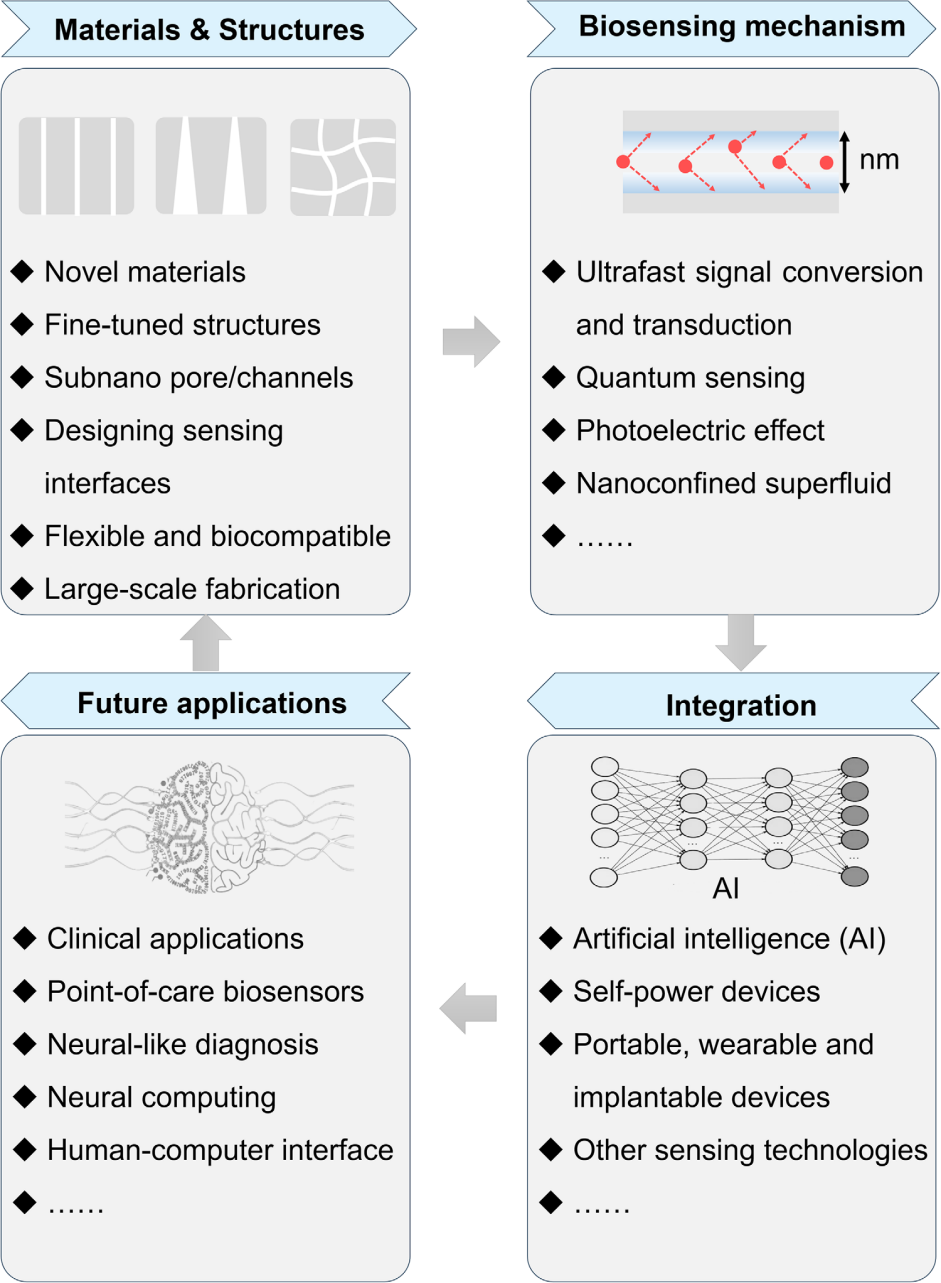
Future challenges and perspectives.

To fully realize the potential of nanofluidic biosensors and translate these advancements into practical applications, several pivotal breakthroughs are required (Figure 6).

### 4.1 Novel Materials and Structures

The performance of nanofluidic biosensors is intrinsically linked to the properties of the materials employed and the precision of their structural design. Novel materials with tailored characteristics and engineered pore/channel architectures are essential for enhancing biosensing capabilities. One promising area of research is the utilization of advanced nanomaterials, such as graphene, carbon nanotubes, and plasmonic nanoparticles, which exhibit unique electrical, optical, and mechanical properties. Semiconductor nanomaterials, for instance, have shown promise in constructing nanofluidic biosensors capable of photoelectric responses. The integration of the photoelectric effect into these devices significantly amplifies signal conversion and conduction efficiency. Moreover, metal‐organic frameworks (MOFs) and covalent organic frameworks (COFs) offer exceptional potential due to their hierarchically customizable pore structures and properties at the molecular level. These materials facilitate precise control over sensor performance at the molecular scale, enabling the development of highly sophisticated nanofluidic biosensors. In addition, the design of the sensing interface is pivotal in optimizing probe‐target interactions. Precise design of the sensing interface and harnessing the synergistic effects between inner walls and outer surfaces of the nanofluidic biosensor would significantly enhance sensitivity and specificity. Additionally, the flexibility and biocompatibility of the construction materials are crucial for subsequent integration and implantation. The use of materials amenable to large‐scale fabrication is essential for reducing production costs and promoting widespread adoption of nanofluidic biosensors.

### 4.2 Innovative Biosensing Mechanisms

The advancement of nanofluidic biosensors necessitates the development of innovative sensing mechanisms to enhance sensitivity, selectivity, and accuracy. A fundamental aspect of this endeavor involves exploring ultrafast ion and molecule transport phenomena within confined nanoscale environments. Achieving rapid and efficient signal conversion and conduction is crucial for optimizing biosensor performance. Moreover, leveraging the quantum effects arising from the nanofluidic size regime offers promising avenues for developing novel sensing modalities. Quantum sensing based on nanofluidic biosensors presents an intriguing possibility. By harnessing quantum effects, these devices may exhibit unprecedented sensitivity and specificity. Semiconductor‐based nanofluidic biosensors, which capitalize on photoelectric effects, introduce complex electron‐ion‐molecule interactions, thereby necessitating innovative approaches to optimize sensor performance. Recent advancements in the theory of nanoconfined superfluidity warrant investigation in the context of nanofluidic biosensors. Exploring the potential for superfluid signal conversion and conduction could lead to the development of groundbreaking biosensing technologies. These research directions collectively aim to push the boundaries of nanofluidic biosensing, enabling the detection and quantification of biomolecules with unparalleled precision and efficiency.

### 4.3 Device Integration of Nanofluidic Biosensors

Miniaturization and integration are paramount in the evolution of biosensor technology, and nanofluidic biosensors, in particular, offer significant potential in these areas due to their early stage of development. As these devices shrink in size and complexity increases, the generation of massive datasets becomes inevitable. Advanced data analytics, including machine learning and deep learning, are indispensable for extracting meaningful insights from these data, thereby enhancing sensor accuracy and expanding application domains. Energy management is another critical factor influencing the practical implementation of biosensors. The development of self‐powered nanofluidic biosensors, capable of harvesting energy from ambient sources such as light, heat, or magnetism, is essential for achieving miniaturization and integration goals. Furthermore, these external energy inputs can be harnessed to modulate fluid transport and biomolecular interactions within the nanofluidic channel, optimizing sensor performance. The convergence of nanofluidic technology with portable, wearable, and implantable platforms holds immense promise for real‐time in situ monitoring and analysis. The intrinsic compatibility of nanofluidic channels with biological systems, owing to their shared ionic environment, offers a distinct advantage over traditional electronic sensors. Additionally, nanofluidic biosensors can serve as versatile platforms for integrating complementary sensing modalities, creating synergistic effects that enhance overall biosensing capabilities. These advancements collectively position nanofluidic biosensors as transformative tools with far‐reaching implications across future fields.

### 4.4 Future Applications

Nanofluidic biosensors hold immense potential for transformative applications. Immediate priorities include enhancing sensitivity, accuracy, precision, and linear range to facilitate rapid translation into clinical and home‐based settings. A biomimetic approach, inspired by the intricacies of the human nervous system, offers a promising avenue for advancing nanofluidic biosensors. By emulating the functionality of neural synapses, these devices can revolutionize clinical diagnosis, precision medicine, and beyond. The development of bioinspired components such as rectifiers, transistors, and memristors, analogous to biological pores, is crucial for expanding the capabilities of nanofluidic biosensors. Moreover, integrating nanofluidic biosensors into bionic synaptic devices can propel advancements in neural computing and information sensing. Addressing the limitations of traditional electronic biosensors, nanofluidic technology presents an opportunity to create novel human–computer interfaces. Leveraging the inherent compatibility between nanofluids and biological systems can significantly enhance human‐computer interaction efficiency, particularly in the context of the burgeoning artificial intelligence landscape.

Overall, biosensing with nanofluidics holds immense promise for revolutionizing healthcare and beyond. By integrating innovative materials, structures, and sensing mechanisms, researchers focusing on nanofluidic biosensors are developing highly sensitive and specific biosensing platforms. Miniaturization, integration, and data analytics are crucial for translating these technologies into practical applications. Overcoming technical challenges through interdisciplinary collaboration is essential to fully realize the potential of nanofluidic biosensors and improve human health.

## Conflicts of Interest

The authors declare no conflicts of interest.
